# Age of ovarian cancer diagnosis among *BRIP1*, *RAD51C*, and *RAD51D* mutation carriers identified through multi-gene panel testing

**DOI:** 10.1186/s13048-021-00809-w

**Published:** 2021-04-29

**Authors:** Shelly Cummings, Susana San Roman, Jennifer Saam, Ryan Bernhisel, Krystal Brown, Johnathan M. Lancaster, Lydia Usha

**Affiliations:** 1 Myriad Genetics Inc., Salt Lake City, UT USA; 2 The Rush Cancer Institute, Rush University, 1725 W. Harrison St. #309, Chicago, IL 60612 USA

**Keywords:** Ovarian cancer, Pan-cancer panel, Genetic testing, Hereditary ovarian cancer

## Abstract

**Background:**

Professional society guidelines recommend risk-reducing salpingo-oophorectomy (RRSO) for women with pathogenic variants (PVs) in ovarian cancer-risk genes. Personalization of that intervention is based on gene-specific phenotypes; however, the age of ovarian cancer diagnosis in women with PVs in moderate penetrance ovarian cancer-risk genes is not well characterized.

Women who had hereditary cancer panel testing from September 2013–May 2019 were included (*N* = 631,950). Clinical/demographic information was compared for women with a PV in *BRIP1, RAD51C,* or *RAD51D* versus in *BRCA1* or *BRCA2.*

**Results:**

PVs in *BRIP1, RAD51C,* or *RAD51D* were identified in 0.5% of all tested women but in 1.6% of women with a history of ovarian cancer (~ 3-fold increase). PVs in *BRCA1* or *BRCA2* were identified in 2.4% of all tested women but in 6.1% of women with a history of ovarian cancer (~ 2.5-fold increase). The proportion of women with a personal or family history of ovarian cancer was similar among women with a PV in *BRIP1*, *RAD51C*, *RAD51D*, *BRCA1*, or *BRCA2*. The median age at ovarian cancer diagnosis was 53 years for *BRCA1*, 59 years for *BRCA2*, 65 years for *BRIP1*, 62 years for *RAD51C*, and 57 years for *RAD51D*.

**Conclusions:**

These data reinforce the importance of identifying PVs in moderate penetrance ovarian cancer-risk genes. The age at ovarian cancer diagnosis was older for women with PVs in *BRIP1, RAD51C,* or *RAD51D*, suggesting that it is safe to delay RRSO until age 45–50 in *RAD51D* PV carriers and possibly until age 50–55 in *BRIP* and *RAD51C* PV carriers.

## Background

Ovarian cancer is uncommon yet deadly, with approximately 251,000 cases and 161,000 deaths (4.5% of all deaths in women) occurring each year, globally [1]. The lifetime risk of ovarian cancer is increased among women with Hereditary Breast and Ovarian Cancer syndrome (HBOC). HBOC is associated with pathogenic variants (PVs) in *BRCA1* or *BRCA2*, where *BRCA1* PVs are associated with a 39–63% lifetime risk of ovarian cancer and *BRCA2* PVs are associated with a 15–27% risk. This is significantly elevated relative to the general population risk of ovarian cancer, which is only 1.3% [2]. More recently, additional genes associated with increased ovarian cancer risk have been identified, including *BRIP1, RAD51C,* and *RAD51D* [3, 4]. These moderate penetrance genes are associated with lower lifetime risks of ovarian cancer than *BRCA1* and *BRCA2*, but still confer significantly increased risk compared to the general population, with lifetime risks ranging from 6 to 15%.

Identification of a PV in an ovarian cancer-risk gene may initiate more intensive and personalized medical management that would not be prompted based on family history alone. National Comprehensive Cancer Network (NCCN) guidelines recommend that women with a PV in *BRCA1* or *BRCA2* consider risk-reducing salpingo-oophorectomy (RRSO) at age 35–45 or earlier, depending on specific family history [5]. These guidelines reflect the level of evidence available regarding the clinical presentation of ovarian cancer in women with PVs in *BRCA1* or *BRCA2*. Specifically, there is robust evidence for a high risk of ovarian cancer at an early age, with *BRCA1* PV carriers having an 8–23% risk of ovarian cancer by age 50 [6–9]. More recent evidence has shown that the risk of early-onset ovarian cancer is lower in *BRCA2* PV carriers (0.4–4%). As such, NCCN guidelines now state that it is reasonable to delay RRSO in *BRCA2* PV carriers until age 40–45.

For women with PVs in *BRIP1*, *RAD51C*, or *RAD51D*, NCCN guidelines recommend that RRSO be considered at age 45–50 [5]. Although there is sufficient evidence of ovarian cancer risk associated with these three genes to justify consideration of RRSO, the guidelines also state that “the current evidence is insufficient to make a firm recommendation as to the optimal age for this procedure” [5]. This reflects the poor understanding of the exact risk of ovarian cancer and the typical age of onset in women with PVs in *BRIP1, RAD51C,* and *RAD51D*. Given the severity of the intervention and associated side-effects, patients and health care providers have strong interest in delaying RRSO until older ages if safe. However, without a better understanding of these parameters, there is uncertainty about the optimal age for surgery and appropriate clinical management of women with PVs in these moderate penetrance ovarian cancer-risk genes.

In order to better characterize the clinical presentation of women with a PV in a moderate penetrance ovarian cancer-risk gene, we evaluated women with a PV in *BRIP1*, *RAD51C*, or *RAD51D* identified during hereditary cancer genetic testing. This includes an assessment of ancestry, personal and family cancer history, and age of ovarian cancer diagnosis*.* In addition, women with PVs in *BRCA1* or *BRCA2* were evaluated for comparison.

## Methods

### Participants

The cohort in this retrospective analysis included women who had testing with a multigene hereditary cancer panel (Myriad Genetic Laboratories, Salt Lake City, UT) between September 2013 and May 2019 (*N* = 631,950). All patients provided informed consent for genetic testing. All patient data was de-identified for analysis. Patients were excluded from this analysis if they were from a state with laws preventing the use of de-identified genetic data for research. Patients were also excluded if they had an unspecified personal cancer history or previous hereditary cancer genetic testing, including founder mutation testing and testing for a known familial mutation. Individuals were also excluded if they were found to have PVs in multiple genes.

### Multi-gene hereditary cancer panel testing and variant classification

Testing was performed in a Clinical Laboratory Improvement Amendments- (CLIA) and College of American Pathology (CAP)-approved laboratory. The hereditary cancer panel was comprised of 25–28 cancer-predisposition genes, including *BRCA1*, *BRCA2*, *BRIP1*, *RAD51C*, and *RAD51D*. This Next Generation Sequencing (NGS) assay has been detailed previously [10, 11]. Sequencing and large rearrangement analysis was performed for all genes evaluated here.

Variant classification was based on guidelines from the American College of Molecular Genetics and Genomics and Association for Molecular Pathology using all available functional, statistical, segregation, and literature evidence, as previously described [12, 13]. Variants with a laboratory classification of Deleterious or Suspected Deleterious were considered pathogenic. This analysis was based on the classification of all variants as of May 2019, regardless of whether they were classified differently when the test report was issued.

### Statistical analysis

The prevalence of PVs in *BRIP1*, *RAD51C*, *RAD51D*, *BRCA1*, or *BRCA2* was evaluated for the full testing cohort as well as the subset of women who had a personal history of ovarian cancer. The clinical presentation of women with PVs in the moderate penetrance ovarian cancer-risk genes (*BRIP1*, *RAD51C*, or *RAD51D*) was evaluated. This included an evaluation of self-reported ancestry, personal and family history of ovarian cancer, and age at diagnosis. Clinical and demographic data were obtained from the provider-completed test request form. Family cancer history was limited to first- and second-degree relatives. History of ovarian cancer included fallopian tube, peritoneal, and ovarian cancer. Analyses were also performed for women with PVs in *BRCA1* and *BRCA2* and women who were tested and found to carry no PV in any gene (PV-negative) for comparison.

Statistical analyses were performed using SAS® software (SAS Institute Inc., Cary, North Carolina, USA) and R software (R Foundation for Statistical Computing, Vienna, Austria).

## Results

### Prevalence of pathogenic variants in ovarian cancer-risk genes

Here, we assessed women who carried a PV in one of five ovarian cancer risk-genes. Overall, 0.5% (3089/631,950) of women tested with the multi-gene panel had a PV in a moderate penetrance ovarian cancer-risk gene. This included 1779 (0.3%) women with a PV in *BRIP1*, 855 (0.1%) women with a PV in *RAD51C*, and 455 (0.1%) women with a PV in *RAD51D* (Table 1). An additional 15,054 (2.4%) women in the testing cohort had a PV in *BRCA1* or *BRCA2* (1.1% for *BRCA1*, 1.3% for *BRCA2*; Table 1). Most of the testing cohort was negative for a PV in any gene (93.7%; 592,309/631,950).

**Table 1:** Demographics and cancer history according to gene

Characteristic	***BRIP1***	***RAD51C***	***RAD51D***	***BRCA1***	***BRCA2***	PV-Negative
**Full Testing Cohort (*N*** **= 631,950)**						
N	1779	855	455	7114	7940	592,309
% of Full Testing Cohort	0.3%	0.1%	0.1%	1.1%	1.3%	93.7%
**Subset of Women with Ovarian Cancer (*N*** **= 27,915)**						
N	233	149	74	975	723	24,468
% of Women with Ovarian Cancer	0.8%	0.5%	0.3%	3.5%	2.6%	88.7%
**Self-Reported Ancestry, N (% of PV Carriers)**						
White/Non-Hispanic^a^	1165 (0.3%)	492 (0.1%)	245 (0.1%)	3863 (1.0%)	4514 (1.2%)	354,404 (93.6%)
Asian	24 (0.2%)	24 (0.2%)	28 (0.2%)	249 (1.7%)	270 (1.9%)	13,577 (93.1%)
Black/African	110 (0.2%)	82 (0.2%)	55 (0.1%)	645 (1.2%)	799 (1.5%)	48,960 (94.4%)
Hispanic/Latino	99 (0.2%)	80 (0.2%)	29 (0.1%)	854 (1.8%)	662 (1.4%)	44,276 (93.4%)
Other^b^	37 (0.3%)	12 (0.1%)	13 (0.1%)	131 (1.1%)	142 (1.2%)	11,595 (94.3%)
Multiple	80 (0.2%)	51 (0.2%)	23 (0.1%)	352 (1.0%)	399 (1.2%)	31,894 (94.2%)
None Specified	264 (0.3%)	114 (0.1%)	62 (0.1%)	1020 (1.1%)	1154 (1.2%)	87,603 (93.8%)
**History of Ovarian Cancer, N (% of PV Carriers)**						
Personal History of Ovarian Cancer	233 (13.1%)	149 (17.4%)	74 (16.3%)	975 (13.7%)	723 (9.1%)	24,768 (4.2%)
Family History of Ovarian Cancer	609 (34.2%)	283 (33.1%)	157 (34.5%)	2496 (35.1%)	2202 (27.7%)	169,680 (28.6%)

Abbreviation: *PV* pathogenic variant

^a^Includes White/Non-Hispanic, Ashkenazi Jewish, and any combination of the two ancestries

^b^Includes Middle Eastern, Native American, and Other

When the subset of women with a personal history of ovarian cancer was considered, the prevalence of PVs in each ovarian cancer-risk gene evaluated here increased, as expected (Table 1). Specifically, 1.6% (456/27,915) of women with a personal history of ovarian cancer had a PV in a moderate penetrance ovarian cancer-risk gene (Table 1). This represents a three-fold increase relative to the full testing cohort. PVs in *BRIP1* were identified in 0.8% (233/27,915) of women with ovarian cancer. PVs in *RAD51C* and *RAD51D* were identified in 0.5% (149/27,915) and 0.3% (74/27,915) of women with ovarian cancer, respectively. The prevalence of PVs in *BRCA1* or *BRCA2* more than doubled within the subset of tested women with a history of ovarian cancer, with a combined prevalence of 6.1% (3.5% for *BRCA1*, 2.6% for *BRCA2*).

The proportion of PV carriers was evaluated for each gene within the most commonly reported ancestries. For the moderate penetrance ovarian cancer-risk genes, the prevalence ranged from 0.1 to 0.3% with no substantial differences by ancestry (Table 1). In comparison, the PV prevalence for *BRCA1* and *BRCA2* ranged from 1.0 to 1.5% in most ancestries (Table 1). Increased prevalence was observed for *BRCA1* among individuals of Asian (1.7%) or Hispanic/Latino (1.8%) ancestry and for *BRCA2* among individuals of Asian ancestry (1.9%). There were no substantial differences in the proportion of individuals who were PV-negative by ancestry (93.1–94.4%; Table 2).

**Table 2:** Age at Ovarian Cancer Diagnosis

Characteristic	***BRIP1***	***RAD51C***	***RAD51D***	***BRCA1***	***BRCA2***	PV-Negative
N^a^	222	144	71	919	690	23,685
Median (years)	65	62	57	53	59	59
IQR	58, 72	54, 69	51, 68	47, 60	52, 67	48, 68
Diagnosed > 50 Years, N (%)	200 (90.1%)	119 (82.6%)	55 (77.5%)	564 (60.7%)	555 (80.4%)	16,383 (69.2%)

Abbreviations: *IQR* Interquartile rage, *PV* pathogenic variant

^a^Only includes patients who specified age at ovarian cancer diagnosis

### Personal and family history of ovarian cancer by gene

The proportion of PV carriers with a personal history of ovarian cancer was evaluated by gene (Table 1). A personal history of ovarian cancer was most common among women with a PV in *RAD51C* (17.4%, 149/855) or *RAD51D* (16.3%, 74/455). In addition, 13.1% (233/1779) of women with a PV in *BRIP1* had a personal history of ovarian cancer. The prevalence of ovarian cancer among women with PVs in these moderate penetrance ovarian-cancer risk genes was similar to *BRCA1*, where 13.7% (975/7114) of *BRCA1* PV carriers had a personal history of ovarian cancer. The prevalence of ovarian cancer was lower among women with PVs in *BRCA2* (9.1%, 723/7940). In comparison, 4.4% of PV-negative women had a personal history of ovarian cancer. This reflects the elevated risk observed within this hereditary cancer testing population relative to a general population.

The proportion of PV carriers who had a first- or second-degree family member with a history of ovarian cancer was also evaluated by gene (Table 1). A family history of ovarian cancer was reported by 34.2% (609/1779) of women with a PV in *BRIP1*, 33.1% (283/855) of women with a PV in *RAD51C*, and 34.5% (157/455) of women with a PV in *RAD51D*. This was similar to what was reported for *BRCA1*, where 35.1% (2496/7114) of carriers had a family history of ovarian cancer. There was a lower prevalence of ovarian cancer in the family for *BRCA2* carriers (27.7%; 2202/7940). This was similar to what was observed among PV-negative women, where 28.6% had a family history of ovarian cancer.

### Age of ovarian cancer by gene

The age at ovarian cancer diagnosis was evaluated according to gene (Table 2). Women with a PV in *BRCA1* had the lowest median age at ovarian cancer diagnosis, at 53 years of age. Women with a PV in *BRCA2* had a median age at diagnosis of 59 years. Similar to *BRCA2*, the median age of ovarian cancer diagnosis was older for women with a PV in *BRIP1* (65 years), *RAD51C* (62 years), or *RAD51D* (57 years). For comparison, PV-negative women with ovarian cancer had a median age at diagnosis of 59 years. This is similar to the median age at ovarian cancer diagnosis in the general population of 63 years [2].

**Fig. 1:**
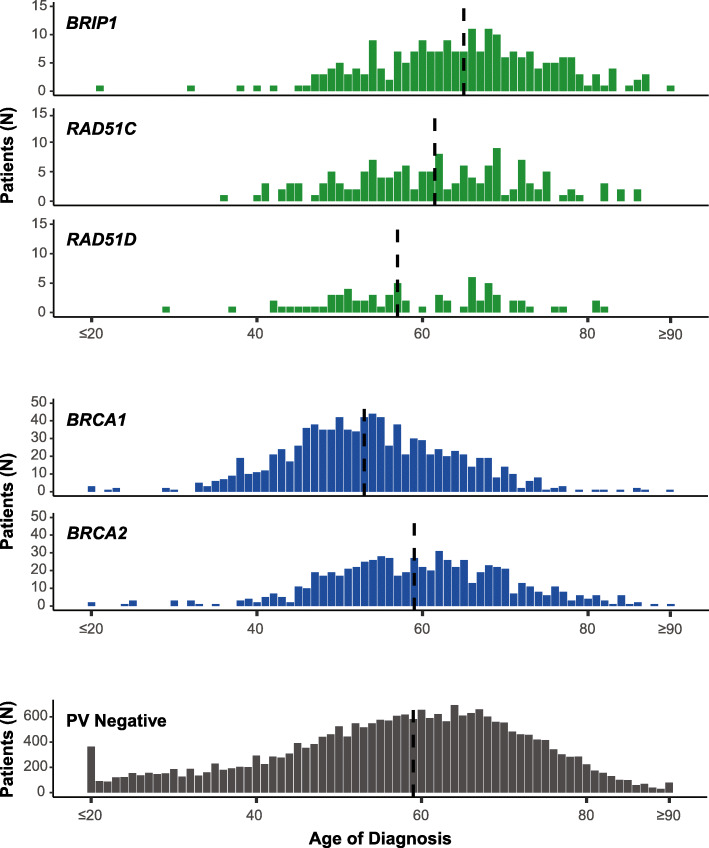
Distribution of age at ovarian cancer diagnosis by gene. Vertical dotted line represents median age. Patients 20 and younger and 90 and older were grouped together

Because the age at ovarian cancer diagnosis may inform management, we also looked at the overall distribution of the age at ovarian cancer diagnosis by gene. Overall, the distribution of age at ovarian cancer diagnosis was skewed to younger ages for women with PVs in *BRCA1* (Fig. 1). This is the only gene where the interquartile range overlapped with age 50 (a proxy for the average age of menopause). Overall, 60.7% (564/919) of women with a PV in *BRCA1* and a personal history of ovarian cancer were diagnosed after age 50, which is similar to what is seen in the general population [2].

In comparison, the distribution of age at diagnosis was skewed to older ages for the other genes evaluated (Fig. 1). For *BRCA2* PV carriers with ovarian cancer, 80.4% (555/690) were diagnosed after age 50. This is similar to what was observed for *BRIP1* (90.1%, 200/222), *RAD51C* (82.6%, 119/144), and *RAD51D* (77.5%, 55/71) (Table 1). In addition, there were very few women with PVs in the moderate penetrance ovarian cancer-risk genes who were diagnosed at very young ages (Fig. 1). The percentage of PV-negative women who had a diagnosis of ovarian cancer after the age of 50 was 69.2% (16,383/23,685).

## Discussion

Ovarian cancer represents 3.7% of all female cancers and is usually diagnosed in advanced stages with a poor prognosis, with overall survival being the worst of all gynecologic malignancies. Professional society guidelines include gene-specific risk reducing recommendations [5]. While these guidelines incorporate decades of evidence for *BRCA1* and *BRCA2*, guidelines are not as clear for other, less well-characterized genes associated with increased ovarian cancer risk. In this analysis, we evaluated the clinical presentation of over 3000 women with PVs in *BRIP1*, *RAD51C*, or *RAD51D* identified by a multigene hereditary cancer panel. To our knowledge, this is the largest published study evaluating the ovarian cancer risk and age of onset associated with pathogenic variants in these moderate penetrance ovarian cancer-risk genes*.*

The data presented here for *BRIP1*, *RAD51C*, and *RAD51D* supports previous research demonstrating an increased risk of ovarian cancer for women with PVs in these genes. The prevalence of a personal or family history of ovarian cancer among women with PVs in *BRIP1*, *RAD51C*, or *RAD51D* was similar to that observed for women with PVs in *BRCA1* or *BRCA2* in this cohort. This supports a recent study that utilized a large clinical cohort to quantify gene-specific ovarian cancer risk. In this study, Kurian et al. demonstrated that *BRIP1*, *RAD51C*, and *RAD51D* are all significantly associated with ovarian cancer [14]. Furthermore, the relative risk of ovarian cancer associated with *RAD51C* and *RAD51D* was comparable to *BRCA2*, with odds ratios for all three genes of approximately five [14]. In addition, there was a substantial enrichment of PVs in these three genes among women with ovarian cancer compared to PV-negative women in this cohort. Collectively, this reiterates the importance of pan-cancer panel testing in women with ovarian cancer. Given the poor prognosis associated with this disease, identifying PVs in genes that confer an increased risk for ovarian cancer outside of *BRCA1* and *BRCA2* is critical for appropriate patient management.

NCCN guidelines recommend that women with PVs in ovarian cancer-risk genes consider RRSO [5]. Given the psychological and medical complications of premature menopause, patients and providers must balance the timing of RRSO with the risk of ovarian cancer. For *BRCA1*, the risk of ovarian cancer at an early age has been well established. This was also observed here, where women with PVs in *BRCA1* had the youngest median age at diagnosis. The median age at ovarian cancer diagnosis of women with a PV in *BRIP1*, *RAD51C*, or *RAD51D* was much older and more than three quarters of women with a PV in one of these three genes and a history of ovarian cancer was diagnosed after the age of 50. For *BRIP1* and *RAD51C*, the median age at ovarian cancer diagnosis was after 60 years. This is comparable to what is seen in the general population, where about half of the women who are diagnosed with ovarian cancer are 63 years or older [2].

At the individual gene level in this cohort, one may determine that it is reasonable to delay RRSO until age 45–50 for women with a PV in a moderate penetrance ovarian cancer-risk gene. In addition, it may be reasonable to delay RRSO until age 50–55 for women with a *BRIP1* or *RAD51C* PV, which is at a time when natural menopause typically occurs. Delayed RRSO in these women may minimize the vasomotor symptoms and cardiovascular risk associated with a premature menopause as well as its negative effect on bone metabolism, and possibly, cognition and longevity [15–17]. Overall, these data aid in supporting providers and their patients in the clinical decision-making process based on a more refined risk of ovarian cancer.

The data presented here also spur an interesting possible application of panel testing among women with ovarian cancer as a method to tailor treatment. Women with defects in the homologous recombination repair (HRR) pathway are more likely to benefit from DNA-damaging therapies, such as PARP inhibitors or platinum-based regimens [18, 19]. Previous research has shown that the presence of germline or tumor PVs in *BRCA1* or *BRCA2* predict benefit from such therapies among women with ovarian cancer [20, 21]. The presence of PVs in other genes in the HRR pathway, including *BRIP1*, *RAD51C*, or *RAD51D*, express a phenotype similar to *BRCA*-related HRR defects [22]. This suggests that panel testing may help guide treatment selection for women with ovarian cancer by identifying PVs in *BRIP1*, *RAD51C*, or *RAD51D* [23].

While this study is informative, it is not without limitations. First, family history information was obtained from provider-completed test request forms and may not be comprehensive. Given the size of this cohort, it was not feasible to confirm the reported family and personal historiesy. In accordance with other data and to help minimize the impact of inaccuracies [24], family history was only considered for first- and second-degree relatives. In addition, our population was composed of women referred for genetic testing and is therefore enriched for individuals with a personal and family history of ovarian cancer. In order to avoid over-interpretation of the data for *BRIP1*, *RAD51C*, and *RAD51D*, we evaluated PV-negative women to provide an appropriate baseline for this elevated risk population. This characteristic should be considered when generalizing this study’s results.

## Conclusion

As hereditary cancer risk assessment is increasingly incorporated into clinical care, clinicians may identify more patients who carry mutations in ovarian cancer risk genes beyond *BRCA1* and *BRCA2*. The data presented here refines our understanding of the ovarian cancer risk and the typical age of diagnosis in women with PVs in moderate penetrance ovarian cancer-risk genes to inform safe and appropriate medical management. Our findings suggest that it is safe to delay RRSO until age 45–50 for women with PVs in *RAD51D* and possibly later for women with PVs in *BRIP1* or *RAD51C*. Overall, these data reiterate the importance of identifying PVs in these genes, given the elevated risk of ovarian cancer in the proband and the family. By more precisely understanding gene-specific ovarian cancer risk, patients and providers can better personalize preventative and treatment interventions.

## Data Availability

The datasets generated and/or analyzed during the current study are not publicly available due patient privacy concerns, but are available from the corresponding author on reasonable request.

## References

[1] Fitzmaurice C, Allen C, Barber RM, Barregard L, Bhutta ZA, Global Burden of Disease Cancer C (2017). Global, Regional, and National Cancer Incidence, Mortality, Years of Life Lost, Years Lived With Disability, and Disability-Adjusted Life-years for 32 Cancer Groups, 1990 to 2015: A Systematic Analysis for the Global Burden of Disease Study. JAMA Oncol.

[2] Cancer Stat Facts: Ovarian Cancer,” Surveillance, Epidemiology, and End Results Program. Available: https://seer.cancer.gov/statfacts/html/ovary.html. Accessed 18 July 2019.

[3] Walsh T, Casadei S, Lee MK, Pennil CC, Nord AS, Thornton AM, Roeb W, Agnew KJ, Stray SM, Wickramanayake A, Norquist B, Pennington KP, Garcia RL, King MC, Swisher EM (2011). Mutations in 12 genes for inherited ovarian, fallopian tube, and peritoneal carcinoma identified by massively parallel sequencing. Proc Natl Acad Sci U S A.

[4] Liliac L, Amalinei C, Balan R, Grigoras A, Caruntu ID (2012). Ovarian cancer: insights into genetics and pathogeny. Histol Histopathol.

[5] Daly MB, Pilarski R, Berry MP, Buys SS, Dickson P, Domchek SM, et al. NCCN Clinical Practice Guidelines in Oncology Genetic/Familial High-Risk Assessment: Breast, Ovarian, and Pancreatic (Version 1.2020) 2020. Available from: https://www.nccn.org/professionals/physician_gls/pdf/genetics_screening.pdf [updated December 4, 2019].

[6] Chen S, Iversen ES, Friebel T, Finkelstein D, Weber BL, Eisen A, Peterson LE, Schildkraut JM, Isaacs C, Peshkin BN, Corio C, Leondaridis L, Tomlinson G, Dutson D, Kerber R, Amos CI, Strong LC, Berry DA, Euhus DM, Parmigiani G (2006). Characterization of BRCA1 and BRCA2 mutations in a large United States sample. J Clin Oncol.

[7] Mavaddat N, Peock S, Frost D, Ellis S, Platte R, Fineberg E, Evans DG, Izatt L, Eeles RA, Adlard J, Davidson R, Eccles D, Cole T, Cook J, Brewer C, Tischkowitz M, Douglas F, Hodgson S, Walker L, Porteous ME, Morrison PJ, Side LE, Kennedy MJ, Houghton C, Donaldson A, Rogers MT, Dorkins H, Miedzybrodzka Z, Gregory H, Eason J, Barwell J, McCann E, Murray A, Antoniou AC, Easton DF, on behalf of EMBRACE (2013). Cancer risks for BRCA1 and BRCA2 mutation carriers: results from prospective analysis of EMBRACE. J Natl Cancer Inst.

[8] Easton DF, Ford D, Bishop DT (1995). Breast and ovarian cancer incidence in BRCA1-mutation carriers. Breast Cancer linkage consortium. Am J Hum Genet.

[9] Kuchenbaecker KB, Hopper JL, Barnes DR, Phillips KA, Mooij TM, Roos-Blom MJ, Jervis S, van Leeuwen FE, Milne RL, Andrieu N, Goldgar DE, Terry MB, Rookus MA, Easton DF, Antoniou AC, McGuffog L, Evans DG, Barrowdale D, Frost D, Adlard J, Ong KR, Izatt L, Tischkowitz M, Eeles R, Davidson R, Hodgson S, Ellis S, Nogues C, Lasset C, Stoppa-Lyonnet D, Fricker JP, Faivre L, Berthet P, Hooning MJ, van der Kolk LE, Kets CM, Adank MA, John EM, Chung WK, Andrulis IL, Southey M, Daly MB, Buys SS, Osorio A, Engel C, Kast K, Schmutzler RK, Caldes T, Jakubowska A, Simard J, Friedlander ML, McLachlan SA, Machackova E, Foretova L, Tan YY, Singer CF, Olah E, Gerdes AM, Arver B, Olsson H, and the BRCA1 and BRCA2 Cohort Consortium (2017). Risks of breast, ovarian, and contralateral breast Cancer for BRCA1 and BRCA2 mutation carriers. JAMA..

[10] Judkins T, Leclair B, Bowles K, Gutin N, Trost J, McCulloch J, Bhatnagar S, Murray A, Craft J, Wardell B, Bastian M, Mitchell J, Chen J, Tran T, Williams D, Potter J, Jammulapati S, Perry M, Morris B, Roa B, Timms K (2015). Development and analytical validation of a 25-gene next generation sequencing panel that includes the BRCA1 and BRCA2 genes to assess hereditary cancer risk. BMC Cancer.

[11] Yurgelun MB, Allen B, Kaldate RR, Bowles KR, Judkins T, Kaushik P (2015). Identification of a Variety of Mutations in Cancer Predisposition Genes in Patients With Suspected Lynch Syndrome. Gastroenterology.

[12] Eggington JM, Bowles KR, Moyes K, Manley S, Esterling L, Sizemore S, Rosenthal E, Theisen A, Saam J, Arnell C, Pruss D, Bennett J, Burbidge LA, Roa B, Wenstrup RJ (2014). A comprehensive laboratory-based program for classification of variants of uncertain significance in hereditary cancer genes. Clin Genet.

[13] Richards S, Aziz N, Bale S, Bick D, Das S, Gastier-Foster J (2015). Standards and guidelines for the interpretation of sequence variants: a joint consensus recommendation of the American College of Medical Genetics and Genomics and the Association for Molecular Pathology. Genet Med.

[14] Kurian AW, Hughes E, Handorf EA, Gutin A, Allen B, Hartman A-R (2017). Breast and ovarian Cancer penetrance estimates derived from Germline multiple-gene sequencing results in women. JCO Prec Oncol.

[15] Parker WH, Broder MS, Chang E, Feskanich D, Farquhar C, Liu Z, Shoupe D, Berek JS, Hankinson S, Manson JAE (2009). Ovarian conservation at the time of hysterectomy and long-term health outcomes in the nurses' health study. Obstet Gynecol.

[16] Jacoby VL, Grady D, Wactawski-Wende J, Manson JE, Allison MA, Kuppermann M, Sarto GE, Robbins J, Phillips L, Martin LW, O'Sullivan MJ, Jackson R, Rodabough RJ, Stefanick ML (2011). Oophorectomy vs ovarian conservation with hysterectomy: cardiovascular disease, hip fracture, and cancer in the Women's Health Initiative observational study. Arch Intern Med.

[17] Rocca WA, Gazzuola Rocca L, Smith CY, Grossardt BR, Faubion SS, Shuster LT, Kirkland JL, Stewart EA, Miller VM (2017). Bilateral oophorectomy and accelerated aging: cause or effect?. J Gerontol A Biol Sci Med Sci.

[18] Coleman RL, Oza AM, Lorusso D, Aghajanian C, Oaknin A, Dean A, Colombo N, Weberpals JI, Clamp A, Scambia G, Leary A, Holloway RW, Gancedo MA, Fong PC, Goh JC, O'Malley DM, Armstrong DK, Garcia-Donas J, Swisher EM, Floquet A, Konecny GE, McNeish IA, Scott CL, Cameron T, Maloney L, Isaacson J, Goble S, Grace C, Harding TC, Raponi M, Sun J, Lin KK, Giordano H, Ledermann JA, Buck M, Dean A, Friedlander ML, Goh JC, Harnett P, Kichenadasse G, Scott CL, Denys H, Dirix L, Vergote I, Elit L, Ghatage P, Oza AM, Plante M, Provencher D, Weberpals JI, Welch S, Floquet A, Gladieff L, Joly F, Leary A, Lortholary A, Lotz J, Medioni J, Tredan O, You B, el-Balat A, Hänle C, Krabisch P, Neunhöffer T, Pölcher M, Wimberger P, Amit A, Kovel S, Leviov M, Safra T, Shapira-Frommer R, Stemmer S, Bologna A, Colombo N, Lorusso D, Pignata S, Sabbatini RF, Scambia G, Tamberi S, Zamagni C, Fong PC, O'Donnell A, Gancedo MA, Herraez AC, Garcia-Donas J, Guerra EM, Oaknin A, Palacio I, Romero I, Sanchez A, Banerjee SN, Clamp A, Drew Y, Gabra HG, Jackson D, Ledermann JA, McNeish IA, Parkinson C, Powell M, Aghajanian C, Armstrong DK, Birrer MJ, Buss MK, Chambers SK, Chen LM, Coleman RL, Holloway RW, Konecny GE, Ma L, Morgan MA, Morris RT, Mutch DG, O'Malley DM, Slomovitz BM, Swisher EM, Vanderkwaak T, Vulfovich M (2017). Rucaparib maintenance treatment for recurrent ovarian carcinoma after response to platinum therapy (ARIEL3): a randomised, double-blind, placebo-controlled, phase 3 trial. Lancet..

[19] Fong PC, Boss DS, Yap TA, Tutt A, Wu P, Mergui-Roelvink M, Mortimer P, Swaisland H, Lau A, O'Connor MJ, Ashworth A, Carmichael J, Kaye SB, Schellens JHM, de Bono JS (2009). Inhibition of poly(ADP-ribose) polymerase in tumors from BRCA mutation carriers. N Engl J Med.

[20] Kim G, Ison G, McKee AE, Zhang H, Tang S, Gwise T (2015). FDA approval summary: Olaparib Monotherapy in patients with deleterious Germline BRCA-mutated advanced ovarian Cancer treated with three or more lines of chemotherapy. Clin Cancer Res.

[21] Mirza MR, Monk BJ, Herrstedt J, Oza AM, Mahner S, Redondo A, et al. Niraparib Maintenance Therapy in Platinum-Sensitive, Recurrent Ovarian Cancer. N Engl J Med. 2016;375(22):2154–216. 10.1056/NEJMoa1611310 27717299

[22] Toss ALC (2013). Molecular mechanisms of PARP inhibitors in BRCA-related ovarian cancer. J Cancer Sci Ther.

[23] Berchuck A, Secord AA, Moss HA, Havrilesky LJ (2017). Maintenance poly (ADP-ribose) polymerase inhibitor therapy for ovarian Cancer: precision oncology or one size fits all?. J Clin Oncol.

[24] Tehranifar P, Wu HC, Shriver T, Cloud AJ, Terry MB (2015). Validation of family cancer history data in high-risk families: the influence of cancer site, ethnicity, kinship degree, and multiple family reporters. Am J Epidemiol.

